# Safety and Recovery Profile of Propofol-Based Intravenous Sedation for Endoscopic Retrograde Cholangiopancreatography in Adults: A Prospective Single-Center Observational Study

**DOI:** 10.7759/cureus.116083

**Published:** 2026-09-10

**Authors:** Muhammad Ahmad Khan, Tariq Mahmood, Mirza Ahmad Raza Baig, Hafsa Noor, Heba Ahmed Aboelfetooh Ahmed

**Affiliations:** 1 Anesthesia, King Khalid Hospital, Hail, SAU; 2 Cardiac Center, King Salman Specialist Hospital, Hail, SAU; 3 Medicine, Liaquat National Hospital, Karachi, PAK; 4 Ophthalmology, King Khalid Hospital, Hail, SAU

**Keywords:** airway management, capnography, endoscopic retrograde cholangiopancreatography, patient satisfaction, procedural sedation, propofol, recovery

## Abstract

Background: Endoscopic retrograde cholangiopancreatography (ERCP) frequently requires deep sedation, but prone positioning, restricted airway access, and variable procedural duration create important safety concerns. We evaluated respiratory and hemodynamic events, recovery, and satisfaction during propofol-based intravenous sedation.

Methods: This prospective, single-center observational study included 100 adults with American Society of Anesthesiologists physical status I or II who underwent elective ERCP for common bile duct stones at King Khalid Hospital in Saudi Arabia from June 2025 to June 2026. Patients received midazolam 2 mg and fentanyl 1 µg/kg intravenously, followed by a propofol infusion of 30-50 µg/kg/minute and 20-30 mg boluses as needed. Patients were prone and received oxygen at 3 L/min through a nasal cannula with an incorporated carbon dioxide sampling line. Standard variables were recorded every five minutes, with continuous waveform capnography. Exact binomial 95% confidence intervals (CIs) were calculated for adverse-event proportions, and Spearman correlation assessed associations with propofol bolus count.

Results: The mean age was 35.1 ± 7.2 years, 53 (53%) were female patients, and 73 (73%) had physical status I. The mean procedure duration was 65.3 ± 12.7 minutes. No oxygen desaturation or tracheal intubation occurred (0/100 (0%); 95% CI, 0-3.6 for each). Airway maneuvers and procedural interruption occurred in the same five patients (5/100 (5%); 95% CI, 1.6-11.3), and postoperative nausea and vomiting (PONV) occurred in four patients (4/100 (4%); 95% CI, 1.1-9.9). Median recovery time was five minutes (interquartile range (IQR), 5-6), and median recovery-room stay was 20 minutes (IQR, 20-20). Mean patient and endoscopist satisfaction scores were 9.63 ± 0.71 and 9.65 ± 0.77, respectively. Longer procedures were associated with more propofol boluses (Spearman ρ = 0.448; p < 0.001), whereas bolus count was not associated with recovery time (ρ = 0.123; p = 0.225).

Conclusion: In this selected, low-risk cohort, propofol-based intravenous sedation with nasal waveform capnography was associated with no recorded hypoxemia or intubation, infrequent airway intervention and PONV, rapid recovery, and high satisfaction. Larger comparative studies using standardized respiratory definitions and prospectively defined capnographic outcomes are warranted.

## Introduction

Endoscopic retrograde cholangiopancreatography (ERCP) is an advanced therapeutic endoscopic procedure used to treat biliary and pancreatic diseases. Compared with routine diagnostic endoscopy, ERCP commonly involves prone or semiprone positioning, restricted airway access, variable procedural stimulation, and prolonged procedure time. Sedation must therefore balance immobility and procedural conditions against respiratory depression, hemodynamic instability, aspiration, and delayed recovery.

Propofol is widely used for advanced endoscopy because of its rapid onset, predictable titration, and short recovery profile. Nevertheless, it has a narrow therapeutic margin and may produce dose-dependent apnea, upper airway obstruction, and hypotension. Recent multidisciplinary consensus supports monitored anesthesia care for many uncomplicated ERCP procedures while reserving general anesthesia with tracheal intubation for patients or procedures with greater aspiration risk, anticipated complexity, gastric outlet obstruction, major bleeding, or other safety concerns [1]. Contemporary sedation guidance also emphasizes patient selection, continuous physiological monitoring, the ability to rescue patients from deeper-than-intended sedation, and immediate access to airway equipment [2,3].

Reported rates of sedation-related events vary because studies use different definitions of hypoxemia, hypotension, airway intervention, sedation depth, and procedural complexity. Anesthesiologist-administered sedation has been associated with high completion and satisfaction, but transient hypoxemia, hypotension, and airway maneuvers remain clinically relevant [4,5]. Alternative regimens using remifentanil, dexmedetomidine, ketamine, remimazolam, or ciprofol aim to reduce propofol exposure or respiratory events, although the optimal technique remains patient- and procedure-dependent [6-12].

The primary objective was to describe the incidence of recorded respiratory and hemodynamic events during propofol-based intravenous sedation in adults undergoing elective ERCP. Secondary objectives were to describe recovery time, recovery-room stay, postoperative nausea and vomiting (PONV), and patient and endoscopist satisfaction. Exploratory objectives were to assess associations between supplemental propofol bolus count and procedure duration, Ramsay Sedation Scale score, recovery, and satisfaction, and to compare selected outcomes between American Society of Anesthesiologists (ASA) physical status I and II patients.

## Materials and methods

Study design and setting

This prospective single-center observational study enrolled consecutive adult patients undergoing elective ERCP under sedation at King Khalid Hospital in Saudi Arabia from June 2025 to June 2026. Reporting was guided by the Strengthening the Reporting of Observational Studies in Epidemiology statement [13].

Participants

The study included 100 adults aged 19-51 years with ASA physical status I or II, as defined by the ASA classification [14], who underwent elective ERCP for common bile duct stones. All patients were evaluated in the anesthesia clinic, completed a preanesthesia assessment, and provided written informed consent for sedation and research participation. The analyzed variables had no missing observations.

Eligibility criteria

Patients were eligible if they were aged 18 years or older, had ASA physical status I or II, were scheduled for elective ERCP for common bile duct stones using planned propofol-based intravenous sedation, could provide informed consent, and had completed a preanesthesia assessment. Patients were excluded if they were younger than 18 years, had ASA physical status III or higher, required emergency ERCP, were pregnant, had an anticipated difficult airway or clinically significant aspiration risk, had severe or uncontrolled cardiac or respiratory disease, had a known allergy or contraindication to propofol, midazolam, or fentanyl, refused or were unable to provide informed consent, or required general anesthesia with endotracheal intubation.

Sedation and monitoring protocol

Baseline vital signs were recorded, and peripheral intravenous access was secured in the preoperative area. In the procedure room, patients were placed prone, and standard monitoring was applied. Pulse rate, noninvasive blood pressure, oxygen saturation, and respiratory rate were documented at five-minute intervals. Oxygen at 3 L/minute was delivered through a nasal cannula incorporating a carbon dioxide sampling line. The capnographic waveform was monitored continuously to facilitate clinical recognition of ventilatory compromise. Because sampling was performed in nonintubated patients, numerical end-tidal carbon dioxide values were interpreted as trends and not as precise surrogates for arterial carbon dioxide tension. All sedation was administered and continuously monitored by a dedicated anesthesiologist experienced in nonoperating-room anesthesia and advanced gastrointestinal endoscopy. The anesthesiologist had no responsibilities related to performing the ERCP and remained exclusively responsible for medication administration, assessment of sedation depth, respiratory and hemodynamic monitoring, and airway or cardiovascular rescue. The endoscopist and endoscopy nursing team did not administer propofol or independently manage sedation.

After standard monitoring had been applied in the procedure room, premedication consisted of midazolam 2 mg and fentanyl 1 µg/kg intravenously. The fixed midazolam dose represented approximately 0.028 mg/kg at the cohort's mean weight of 72.2 kg and was selected as a conservative standard adult dose for anxiolysis and amnesia in this relatively young ASA physical status I-II population. Sedation was then individualized with a propofol infusion at 30-50 µg/kg/minute, titrated to clinical need, with supplemental 20-30 mg propofol boluses as needed. Sedation depth was assessed using the Ramsay Sedation Scale [15]: score 2, cooperative, oriented, and tranquil; score 3, responds to commands only; score 4, brisk response to a light glabellar tap or loud auditory stimulus; and score 5, sluggish response to these stimuli. The number of supplemental boluses and average Ramsay score were recorded. Sedation depth was not separately documented at a standardized time point after midazolam but before fentanyl and propofol; therefore, the independent contribution of midazolam to deeper-than-intended initial sedation could not be determined.

Outcomes and operational definitions

The recorded outcomes were oxygen desaturation, pulse rate increase or decrease by more than 20% of baseline, blood pressure increase or decrease by more than 20% of baseline, airway maneuver, tracheal intubation, procedural interruption, PONV, recovery time, recovery room stay, and satisfaction. Visual analog scale for patient satisfaction and visual analog scale for endoscopist satisfaction were recorded on 0-10 visual analog scales, with higher scores indicating greater satisfaction, consistent with prior ERCP satisfaction assessment [5].

Oxygen desaturation was defined as peripheral oxygen saturation below 90% for at least 10 seconds. An airway maneuver comprised chin lift, jaw thrust, repositioning, suctioning, or insertion of an oral or nasal airway. Tracheal intubation was recorded as a separate severe respiratory outcome.

Pulse rate and noninvasive blood pressure were recorded at baseline and at five-minute intervals. For each variable, the case-report form classified the observed course as within 20% of baseline, more than 20% above baseline, or more than 20% below baseline. These relative categories were not equivalent to prespecified absolute diagnostic thresholds for tachycardia, bradycardia, hypertension, or hypotension. Exact magnitude beyond the 20% threshold, event duration, timestamps, associated symptoms, presumed cause, clinical significance, and cardiovascular treatment were not systematically captured.

Recovery time was defined as the interval from discontinuation of propofol until the patient was awake, responded appropriately to verbal commands, and maintained stable spontaneous ventilation. Recovery-room stay was the total time spent in the recovery area. Discharge required return to baseline consciousness with stable vital signs, satisfactory oxygenation, intact protective reflexes, controlled nausea and pain, and no sedation-related complication. Recovery time and recovery-room stay were separate descriptive outcomes rather than alternative discharge criteria.

Although continuous waveform capnography was used clinically to monitor ventilation trends, apnea, hypoventilation, and airway obstruction were not systematically captured as separate case-level study variables. They were therefore not retained as prespecified analyzable outcomes, and their incidence, duration, and severity could not be reported. The oxygen-saturation threshold was consistent with prospective research on sedation-related complications during ERCP [4].

Sample size rationale

No formal a priori power-based sample-size calculation was performed because the study was designed as a single-center descriptive feasibility study without a comparator group or prespecified hypothesis test. A pragmatic sample of 100 consecutive eligible participants was enrolled during the defined study period to generate preliminary estimates of sedation-related events, recovery, and satisfaction with exact 95% confidence intervals (CIs). This sample could characterize common outcomes but had limited precision for uncommon adverse events and was underpowered for small between-group differences. With zero events among 100 participants, the upper limit of the two-sided exact 95% CI is approximately 3.6%; therefore, an observed count of zero does not establish absence of risk.

Statistical analysis

Continuous variables were summarized as mean ± standard deviation and median with interquartile range (IQR), and categorical variables as number and percentage. Body mass index was calculated as weight in kilograms divided by height in meters squared. Exact Clopper-Pearson 95% CIs were calculated for key safety-event proportions, including zero-event outcomes. Spearman rank correlation assessed relationships between propofol bolus count and procedure duration, average Ramsay score, recovery time, recovery-room stay, and satisfaction. Exploratory comparisons used the Mann-Whitney U test for continuous variables and two-sided Fisher's exact test for categorical variables. Odds ratios (ORs) compared ASA II with ASA I as the reference group. When a 2 x 2 table contained a zero cell, the Haldane-Anscombe correction was applied by adding 0.5 to all four cells before calculating the OR and its 95% CI. Tests were two-sided, and p < 0.05 was considered statistically significant. No multiplicity adjustment was applied because the analyses were exploratory. Analyses were performed using Python 3.12.13 (Python Software Foundation, Beaverton, OR) and SciPy 1.17.0 (SciPy Developers, Austin, TX).

Ethical considerations

The Ethical Committee of King Khalid Hospital, Saudi Arabia, approved the study (approval number 625-01-002316). All participants provided written informed consent for clinical sedation and research participation.

## Results

Participant and procedural characteristics

All 100 records were complete for the analyzed variables. Participants had a mean age of 35.1 ± 7.2 years (range, 19-51 years) and a mean body mass index of 26.9 ± 2.6 kg/m²; 53 (53%) were female and 47 (47%) were male participants; 73 (73%) had ASA physical status I, and 27 (27%) had ASA physical status II. All underwent ERCP for common bile duct stones. Mean procedure duration was 65.3 ± 12.7 minutes. Patients received a median of one supplemental propofol bolus, and 95 (95%) had average Ramsay scores of 2 or 3 (Table 1).

**Table 1:** Participant, procedural, and sedation characteristics (n = 100) Values are mean ± standard deviation, median (IQR), or n (%) ASA: American Society of Anesthesiologists; SD: standard deviation; IQR: interquartile range

Characteristic	Statistic	Value
Age, years	Mean ± SD	35.1 ± 7.2
	Median (IQR)	35 (30-41)
Sex	Female, n (%)	53 (53%)
	Male, n (%)	47 (47%)
Weight, kg	Mean ± SD	72.2 ± 10.2
Height, cm	Mean ± SD	163.6 ± 7.5
Body mass index, kg/m²	Mean ± SD	26.9 ± 2.6
ASA physical status	Class I, n (%)	73 (73%)
	Class II, n (%)	27 (27%)
Procedure duration, minutes	Mean ± SD	65.3 ± 12.7
	Median (IQR)	66.5 (57-75)
Propofol boluses, n	Mean ± SD	1.26 ± 0.97
	Median (IQR)	1 (1-2)
	Range	0-4
Average Ramsay score	Mean ± SD	2.58 ± 0.65
	Median (IQR)	3 (2-3)
Ramsay score	Score 2	49 (49%)
	Score 3	46 (46%)
	Score 4	3 (3%)
	Score 5	2 (2%)

No oxygen desaturation or tracheal intubation was recorded (0/100 (0%) for each; exact 95% CI, 0-3.6). Five patients required an airway maneuver (5/100 (5%); exact 95% CI, 1.6-11.3), and the procedures of the same five patients were temporarily interrupted (5/100 (5%); exact 95% CI, 1.6-11.3). Apnea, hypoventilation, and airway obstruction were not recorded as separate case-level variables; therefore, their incidence, duration, severity, and relationship to the five airway interventions could not be determined. Pulse rate excursions beyond 20% of baseline occurred in 11 patients (11/100 (11%)): increases in eight (8%) and decreases in three (3%). Blood pressure excursions beyond 20% of baseline occurred in the same 11 patients (11/100 (11%)): increases in seven patients (7%) and decreases in four patients (4%). All 11 were ASA II. Seven patients had both pulse rate and blood pressure increases recorded, three had both decreases recorded, and one had a pulse-rate increase with a blood-pressure decrease. Because event timestamps were unavailable, simultaneity could not be established. Exact magnitude beyond the threshold, duration, associated symptoms, presumed cause, clinical significance, and treatment were not available. PONV occurred in four patients (4%) (Table 2).

**Table 2:** Safety, recovery, and satisfaction outcomes Exact Clopper-Pearson CIs are shown for binary events. The five procedural interruptions occurred in the same five patients who required airway maneuvers. The 11 patients with pulse-rate excursions were the same 11 patients with blood-pressure excursions. Event timestamps, exact magnitude beyond the 20% threshold, duration, clinical significance, presumed cause, and cardiovascular treatments were not captured. Apnea, hypoventilation, and airway obstruction were not recorded as separate analyzable outcomes CI: confidence interval; PONV: postoperative nausea and vomiting; VAS-P: visual analog scale for patient satisfaction; VAS-S: visual analog scale for endoscopist satisfaction

Outcome	Statistic	Result	95% CI
Oxygen desaturation	Frequency	0 (0%)	0-3.6
Tracheal intubation		0 (0%)	0-3.6
Airway maneuver		5 (5%)	1.6-11.3
Procedural interruption		5 (5%)	1.6-11.3
Pulse-rate increase >20% from baseline		8 (8%)	3.5-15.2
Pulse-rate decrease >20% from baseline		3 (3%)	0.6-8.5
Any pulse-rate excursion >20%		11 (11%)	5.6-18.8
Blood-pressure increase >20% from baseline		7 (7%)	2.9-13.9
Blood-pressure decrease >20% from baseline		4 (4%)	1.1-9.9
Any blood-pressure excursion >20%		11 (11%)	5.6-18.8
PONV		4 (4%)	1.1-9.9
Recovery time, minutes	Mean ± SD	5.66 ± 1.23	-
	Median (IQR)	5 (5-6)	-
Recovery-room stay, minutes	Mean ± SD	21.03 ± 3.55	-
	Median (IQR)	20 (20-20)	-
Patient satisfaction (VAS-P)	Mean ± SD	9.63 ± 0.71	-
	Median (IQR)	10 (9-10)	-
Endoscopist satisfaction (VAS-S)	Mean ± SD	9.65 ± 0.77	-
	Median (IQR)	10 (10-10)	-

Exploratory associations

Propofol bolus count had a moderate positive association with procedure duration (Spearman ρ = 0.448; p < 0.001). It was not associated with average Ramsay score, recovery time, recovery-room stay, or either satisfaction score (Table 3). In exploratory ASA-class comparisons, ASA II patients received more supplemental propofol boluses than ASA I patients (median 2 (IQR, 1-3) vs. 1 (IQR, 0-1); Mann-Whitney U = 451.0; p < 0.001). All pulse rate and blood pressure excursions beyond 20% of baseline occurred among ASA II patients. For ASA II vs. ASA I, Haldane-Anscombe-corrected ORs were 64.08 (95% CI, 3.54-1159.42; p < 0.001) for pulse rate increase, 21.00 (95% CI, 1.05-421.08; p = 0.018) for pulse-rate decrease, 53.78 (95% CI, 2.95-981.65; p < 0.001) for blood pressure increase, and 28.15 (95% CI, 1.46-542.39; p = 0.004) for blood pressure decrease. These extremely wide CIs reflect sparse events and zero ASA I event cells. Airway-maneuver, procedural-interruption, and PONV rates did not differ detectably by ASA class (all Fisher's exact p = 1.000) (Table 4). These post hoc analyses were underpowered and unadjusted for multiplicity and confounding and should not be interpreted as evidence that ASA II status caused the observed excursions.

**Table 3:** Spearman correlations with supplemental propofol bolus count Spearman ρ is the test statistic. Tests were two-sided; p < 0.05 was considered statistically significant VAS-P: visual analog scale for patient satisfaction; VAS-S: visual analog scale for endoscopist satisfaction

Variable	Spearman ρ	p value
Procedure duration	0.448	<0.001
Average Ramsay score	0.004	0.965
Recovery time	0.123	0.225
Recovery-room stay	0.042	0.676
Patient satisfaction (VAS-P)	-0.061	0.547
Endoscopist satisfaction (VAS-S)	0.181	0.071

**Table 4:** Exploratory comparisons by ASA physical status Values are median (IQR) or n (%). ORs compare ASA II with ASA I as the reference group. For rows containing a zero cell, the Haldane-Anscombe correction added 0.5 to all four cells before calculation of the OR and 95% CI. Categorical p values are from two-sided Fisher's exact tests. The pulse-rate and blood-pressure excursions involved the same 11 ASA II patients: seven had both increases recorded, three had both decreases recorded, and one had a pulse-rate increase with a blood-pressure decrease. Timestamps were unavailable, so simultaneity could not be established. Tests were post hoc, exploratory, and unadjusted for multiplicity or confounding OR: odds ratio; CI: confidence interval; PONV: postoperative nausea and vomiting; IQR: interquartile range; ASA: American Society of Anesthesiologists; VAS-P: visual analog scale for patient satisfaction; VAS-S: visual analog scale for endoscopist satisfaction

Outcome	ASA I (n = 73)	ASA II (n = 27)	Test statistic or OR (95% CI)	p value
Procedure duration, minutes	66 (57-74)	67 (58-75)	U = 936.5	0.706
Propofol boluses, n	1 (0-1)	2 (1-3)	U = 451.0	<0.001
Average Ramsay score	2 (2-3)	3 (2-3)	U = 934.5	0.658
Recovery time, minutes	5 (5-6)	5 (5-6)	U = 986.0	1.000
Recovery room stay, minutes	20 (20-20)	20 (20-20)	U = 998.0	0.892
Patient satisfaction (VAS-P)	10 (9-10)	10 (10-10)	U = 890.5	0.339
Endoscopist satisfaction (VAS-S)	10 (10-10)	10 (10-10)	U = 984.5	0.996
Pulse rate increase >20% from baseline	0 (0%)	8 (29.6%)	OR = 64.08 (3.54-1159.42)	<0.001
Pulse rate decrease >20% from baseline	0 (0%)	3 (11.1%)	OR = 21.00 (1.05-421.08)	0.018
Blood pressure increase >20% from baseline	0 (0%)	7 (25.9%)	OR = 53.78 (2.95-981.65)	<0.001
Blood pressure decrease >20% from baseline	0 (0%)	4 (14.8%)	OR = 28.15 (1.46-542.39)	0.004
Airway maneuver	4 (5.5%)	1 (3.7%)	OR = 0.66 (0.07-6.22)	1.000
Procedural interruption	4 (5.5%)	1 (3.7%)	OR = 0.66 (0.07-6.22)	1.000
PONV	3 (4.1%)	1 (3.7%)	OR = 0.90 (0.09-9.02)	1.000

## Discussion

In this small cohort of 100 relatively young, highly selected ASA I-II adults undergoing elective ERCP, anesthesiologist-administered propofol-based intravenous sedation was accompanied by no recorded oxygen desaturation or tracheal intubation, airway maneuvers and temporary procedural interruption in 5 (5%) patients, PONV in 4 (4%), rapid recovery, and high satisfaction. These observations apply only to this single-center low-risk cohort. The absence of a comparator prevents attribution of the outcomes to propofol, capnography, or anesthesiologist administration, and the findings neither establish overall regimen safety nor support generalization to typical older or high-risk ERCP populations.

The absence of recorded oxygen desaturation should be interpreted alongside the exact 95% CI: with 100 observations, the true event rate remains compatible with values up to 3.6%. It is also sensitive to the threshold and monitoring method. Prospective studies demonstrate that desaturation during ERCP can be clinically important and that incidence varies with event definitions, oxygen strategy, position, and capnography use [5,16]. In this study, patients were prone and received oxygen at 3 L/minute through a nasal cannula with an incorporated carbon dioxide sampling line. Continuous waveform monitoring facilitated clinical recognition of ventilatory compromise. However, apnea, hypoventilation, and airway obstruction were not collected as separate analyzable outcomes. In nonintubated patients, mouth breathing, supplemental oxygen, endoscope position, secretions, and sampling-line displacement can alter the sampled waveform and numerical end-tidal carbon dioxide value. Numerical values should therefore be interpreted as ventilation trends and not as exact estimates of arterial carbon dioxide tension.

The airway-maneuver rate of 5 (5%) was lower than the frequency of minor respiratory interventions reported in some broader cohorts, but the present study excluded patients with ASA physical status III or IV and had a mean age of 35.1 ± 7.2 years, an age range of 19-51 years, and a mean body mass index of 26.9 ± 2.6 kg/m². Zhang et al. found rapid recovery and high satisfaction with anesthesiologist-administered ERCP sedation but still reported events requiring active management in a broader population [4]. De Vico et al. found propofol-remifentanil deep sedation with spontaneous ventilation feasible, although coughing, movement, and occasional hypoxemia occurred [6]. These contrasts emphasize that the zero-desaturation and low-complication findings in the present study are directly tied to a highly selected low-risk demographic and cannot be extrapolated to typical advanced endoscopic practice.

The positive association between procedure duration and propofol bolus count is clinically plausible because longer procedures increase cumulative stimulation and the period for which sedation must be maintained. Bolus count was not correlated with recovery time. This finding should not be interpreted as evidence that repeated boluses are harmless because total propofol dose per kilogram, exact bolus dose, timing of the final dose, infusion adjustments, and cumulative coadministered sedatives were not available. Midazolam was administered as a fixed 2 mg dose, and sedation depth was not separately recorded between midazolam administration and the subsequent fentanyl-propofol regimen; therefore, its independent contribution to deeper-than-intended initial sedation cannot be determined. Future studies should collect complete weight-adjusted cumulative drug doses and time-resolved sedation assessments.

Recent trials have evaluated dexmedetomidine, ketamine, remimazolam, and ciprofol as alternatives or adjuncts intended to preserve ventilation or hemodynamic stability [7-12]. A randomized trial reported less hypoxemia with dexmedetomidine than with propofol during ERCP, although the regimens have different onset, hemodynamic, and recovery profiles [7]. No single regimen is optimal for every patient; contemporary guidance emphasizes risk stratification and the selection of monitored anesthesia care or general anesthesia based on patient and procedural features [1].

Patient and endoscopist satisfaction were high. Satisfaction is clinically relevant because inadequate sedation can lead to movement, interruption, failed cannulation, or repeat procedures. However, satisfaction may be affected by recall, measurement timing, social desirability, procedural outcome, and the identity of the assessor. Future research should use validated questionnaires, prespecified measurement times, and blinded assessment where feasible.

Strengths and limitations

Strengths include prospective data collection, complete observations across 100 cases, simultaneous reporting of respiratory, hemodynamic, recovery, PONV, and satisfaction outcomes, prespecified oxygen desaturation and airway-intervention definitions, continuous waveform capnography for clinical monitoring, standardized recovery assessment, and exact CIs for rare events.

Several limitations require emphasis. First, the single-center observational design without a comparator prevents causal inference or conclusions about comparative effectiveness, superiority, or overall safety. Second, the pragmatic sample of 100 participants was not based on an a priori power calculation, had limited precision for uncommon events, and was underpowered for small group differences. Third, the cohort was exceptionally young and low risk: mean age was 35.1 ± 7.2 years (range, 19-51 years), mean body mass index was 26.9 ± 2.6 kg/m², and eligibility was restricted to ASA physical status I or II. The findings should not be extrapolated to older, frail, obese, multimorbid, ASA physical status III-IV, emergency, or otherwise high-risk ERCP populations. Fourth, pulse rate and blood pressure were retained only as directional categories relative to a 20% baseline threshold; timestamps, exact magnitude, duration, symptoms, clinical significance, presumed cause, and treatment were not captured. Consequently, the data cannot establish simultaneity or diagnoses of tachycardia, bradycardia, hypertension, or hypotension. Fifth, apnea, hypoventilation, and airway obstruction were not available as separate analyzable outcomes despite clinical waveform monitoring; therefore, their incidence, duration, and severity cannot be reported, and the absence of recorded oxygen desaturation should not be interpreted as absence of other respiratory compromise. Sixth, total propofol dose, infusion adjustments, cumulative drug exposure, detailed ERCP complexity grading, cannulation success, and procedure-specific outcomes were unavailable. Sedation depth was not separately recorded after fixed-dose midazolam and before fentanyl and propofol, precluding assessment of midazolam's independent contribution to deeper-than-intended initial sedation. Seventh, nasal carbon dioxide sampling in nonintubated prone patients is vulnerable to dilution and artifact and cannot be assumed to equal arterial carbon dioxide tension. Finally, satisfaction ratings may be vulnerable to measurement and observer bias.

## Conclusions

In this prospective single-center observational study of 100 highly selected, relatively young adults with ASA physical status I or II, anesthesiologist-administered propofol-based intravenous sedation was accompanied by no recorded oxygen desaturation or tracheal intubation, airway intervention in five patients (5%), rapid recovery, and high patient and endoscopist satisfaction. These findings are descriptive and hypothesis-generating. The pragmatic sample was not powered to detect uncommon adverse events, and the absence of a comparator group prevents conclusions regarding causality, comparative effectiveness, superiority, or overall regimen safety.

The results should not be generalized to older, frail, obese, multimorbid, ASA physical status III-IV, emergency, or otherwise high-risk patients undergoing ERCP. Larger, adequately powered, multicenter comparative studies should enroll representative ERCP populations and include standardized respiratory and hemodynamic definitions, absolute event values, duration, severity and treatment, prospectively defined capnographic outcomes, time-resolved and weight-adjusted cumulative sedative dosing, ERCP complexity grading, and explicit criteria for selecting monitored anesthesia care vs. general anesthesia.
